# Analysis of Phosphorus Use Efficiency Traits in *Coffea* Genotypes Reveals *Coffea arabica* and *Coffea canephora* Have Contrasting Phosphorus Uptake and Utilization Efficiencies

**DOI:** 10.3389/fpls.2016.00408

**Published:** 2016-03-31

**Authors:** Ana P. Neto, José L. Favarin, John P. Hammond, Tiago Tezotto, Hilton T. Z. Couto

**Affiliations:** ^1^Departamento de Produção Vegetal, Escola Superior de Agricultura Luiz de Queiroz, Universidade De São PauloPiracicaba, Brazil; ^2^School of Agriculture, Policy and Development and Centre for Food SecurityReading, UK; ^3^Centro Universitário da Fundação Octavio BastosSão João da Boa Vista, Brazil; ^4^Departamento de Recursos Florestais, Escola Superior de Agricultura Luiz de Queiroz, Universidade De São PauloPiracicaba, Brazil

**Keywords:** *Coffea arabica*, *Coffea canephora*, uptake, utilization, efficiency, phosphate, phosphorus

## Abstract

**Background and Aims:** Phosphate (Pi) is one of the most limiting nutrients for agricultural production in Brazilian soils due to low soil Pi concentrations and rapid fixation of fertilizer Pi by adsorption to oxidic minerals and/or precipitation by iron and aluminum ions. The objectives of this study were to quantify phosphorus (P) uptake and use efficiency in cultivars of the species *Coffea arabica* L. and *Coffea canephora* L., and group them in terms of efficiency and response to Pi availability.

**Methods:** Plants of 21 cultivars of *C. arabica* and four cultivars of *C. canephora* were grown under contrasting soil Pi availabilities. Biomass accumulation, tissue P concentration and accumulation and efficiency indices for P use were measured.

**Key Results:** Coffee plant growth was significantly reduced under low Pi availability, and P concentration was higher in cultivars of *C. canephora*. The young leaves accumulated more P than any other tissue. The cultivars of *C. canephora* had a higher root/shoot ratio and were significantly more efficient in P uptake, while the cultivars of *C. arabica* were more efficient in P utilization. Agronomic P use efficiency varied among coffee cultivars and E16 Shoa, E22 Sidamo, Iêmen and Acaiá cultivars were classified as the most efficient and responsive to Pi supply. A positive correlation between P uptake efficiency and root to shoot ratio was observed across all cultivars at low Pi supply. These data identify *Coffea* genotypes better adapted to low soil Pi availabilities, and the traits that contribute to improved P uptake and use efficiency. These data could be used to select current genotypes with improved P uptake or utilization efficiencies for use on soils with low Pi availability and also provide potential breeding material and targets for breeding new cultivars better adapted to the low Pi status of Brazilian soils. This could ultimately reduce the use of Pi fertilizers in tropical soils, and contribute to more sustainable coffee production.

## Introduction

Brazilian coffee producing areas are concentrated in the tropical regions, whose soils are highly weathered with low plant available phosphate (Pi). Thus, Pi fertilizers are necessary to maintain crop production. Fertilizer Pi can be adsorbed by clay minerals or precipitated by iron (Fe^2+^) and aluminum (Al^3+^), which reduces the availability of Pi in tropical soil with only 10–20% of the Pi applied being absorbed by the crop (Mclaughlin et al., 1991; Bolland and Gilkes, 1998; Sousa and Lobato, 2003). Consequently, soil Pi availability can be a significant limiting factor in crop and coffee production (Vance et al., 2003; Lynch, 2007).

There are major concerns about the future availability of non-renewable phosphate rock reserves that could impact on Pi fertilizer availability and costs (Cordell et al., 2009; USGS, 2012). Thus, identification of economic and sustainable approaches that improve the efficiency of Pi fertilizer use are a high priority in crop breeding programs (Parentoni and Junior, 2008; Maia et al., 2011; Wiel et al., 2016). Since coffee breeding in the past has focused on breeding for high yields and pest and disease resistance under high soil fertility, there is scope and need to develop coffee cultivars for the future that are better adapted to low soil Pi availability.

Coffee has a high genetic variability and several studies indicate variation between different genotypes in relation to absorption and translocation of nutrients (Amaral et al., 2011), including zinc and P (Reis and Martinez, 2002), potassium, calcium, magnesium, and sulfur (Tomaz et al., 2003, 2008), boron, zinc, copper, and manganese (Tomaz et al., 2011), but few have explored P use efficiency. In research conducted by Martins et al. (2015), the authors indicated high genotypic variability for *Coffea canephora* genotypes cultivated in environments with low Pi availability in the soil and classified seven of the 13 cultivars studied as tolerant to low soil Pi availability.

Significant genetic variation has previously been observed in phosphorus use efficiency (PUE) related traits in plants (Wissuwa and Ae, 2001; Osborne and Rengel, 2002a,b; Ozturk et al., 2005; Gunes et al., 2006; Hammond et al., 2009). Genetic variation in PUE and related traits has previously been shown to be heritable (Fawole et al., 1982; Hammond et al., 2009) and once these traits have been identified in a cultivar, it could be used in breeding programs to improve crop PUE. Component traits that give rise to improved PUE are also identified through these studies and provide more focused breeding targets to achieve improved crop PUE (Hammond et al., 2009).

There are numerous definitions for PUE (White et al., 2005; Hammond et al., 2009; Rose and Wissuwa, 2012). Phosphorus (P) uptake efficiency refers to the plants ability to obtain Pi from the soil, and P utilization efficiency to the capacity for biomass production using the P absorbed (Wang et al., 2010). Increasing PUE can be achieved either by increasing uptake capacity or by optimizing its utilization (Manske et al., 2001; Shenoy and Kalagudi, 2005; Parentoni and Junior, 2008). The relative importance of each measure depends on the environmental conditions under which the crop is growing. In high input agri-ecosystems, the importance of P uptake efficiency is diminished, given the greater availability of Pi from fertilizer inputs. Improvements, in P utilization efficiency and reductions in the P removed at harvest would be of agronomic benefit under these conditions. In contrast, under low input systems, where soil Pi availability may be low, improvements in P acquisition and utilization are likely to be of benefit.

This study aims to quantify P uptake and use efficiency in cultivars of the species *Coffea arabica* L. and *Coffea canephora* L., group them in terms of efficiency and their response to Pi availability. This will identify genotypes and traits to support future breeding of coffee cultivars for low soil Pi conditions, reducing inputs and improving the sustainability of production.

## Materials and methods

### Plant material

A total of 21 coffee cultivars of the species *Coffea arabica* L. and four cultivars of the species *C. canephora* (Supplementary Table 1) were grown under glasshouse conditions, in Piracicaba (22°42′27.98″ S and 47°37′58.21″ W, altitude 547 m), São Paulo State, Brazil, from July 2010 to August 2011. The materials were selected from the germplasm bank of the Instituto Agronômico de Campinas (IAC). *C. arabica* genetic material introduced to Brazil before the 1970's shows a narrow genetic base with high relationship between cultivars. Therefore, we also included exotic introductions from other countries, selections and botanical forms representing the two main species cultivated.

### Growth conditions

All seeds were germinated in washed sand and irrigated with deionized water. Seedlings were transplanted into 9 dm^3^ pots (one seedling per pot) before the emergence of cotyledons. Pots were filled with soil, classified as Oxisol with medium texture, collected in Piracicaba, São Paulo State, at 20 cm below the surface to eliminate the effect of previous fertilizations (Supplementary Table 2). A low Pi treatment was used consisting of the original Pi concentration in the soil (8 mg Pi dm^−3^ - resin extraction). The resin Pi extraction procedure was based upon ion exchange using an ion exchange resin to measure plant available Pi (Van Raij et al., 1986). A high Pi treatment was obtained by the addition of 3.7 g of monobasic ammonium phosphate (NH_4_H_2_PO_4_) and 1.56 g monobasic potassium phosphate (KH_2_PO_4_) per pot, to give a soil resin extractable Pi concentration of 120 mg Pi dm^−3^, considered high for coffee production (Lani et al., 2007). In treatments without Pi, N and K were added at each pot to ensure that all received the same amount (1.28 g NH_4_NO_3_, 0.89 g K_2_SO_4_, and 0.095 g KCl). The limestone and fertilizers were mixed into the soil for each pot and incubated for 20 days, with moisture maintained at 60% of water retention capacity. Limestone was added to correct soil acidity. The remaining nutrients were provided in the following concentrations (mg dm^−3^): N (${\text{NO}}_{3}^{-}$ and ${\text{NH}}_{4}^{+}$) -50; K-50; S-50; B-1; Co-0.1; Cu-1; Cl-5; Mn-5; Mo-0.15; Ni-0.1 and Zn-3. Soil chemical and physical properties were determined prior to transplanting of seedlings (Supplementary Table 2). Basal N and K applications were supplemented with four applications in solution each month after plant emergence (mg dm^−3^): N-50, K-50, and S-20. Pots were irrigated daily by means drip irrigation and the volume of water was calculated so that there was no runoff of water.

The experimental design consisted of randomized blocks, in a factorial design: 25 (cultivars) × 2 (Pi concentrations: low and high Pi), with five repetitions. Glasshouse average temperature was 23 ± 3.35°C (mean ± SD) and relative humidity 70% ± 12.04 (mean ± SD).

### Plant analysis

Plant height, stem diameter, and number of branches were measured every month. The height was obtained from the base to the apex of the orthotropic branch, and the stem diameter at 2 cm from the stem base. The orthotropic branch is the branch that grows vertically and supports the side branches. At harvest, 9 months after germination, the number of young and mature leaves, leaf area, and the dry mass of young leaves, mature leaves, branches, stem and roots were determined. Mature leaves were considered those fully expanded, with intense green color, and young leaves were considered as still expanding, with a pale green color. Leaf area was determined with a LiCor 3100 leaf area meter (LiCor, Nebraska, USA).

Plant material was rinsed in deionized water. Plagiotropic branches stem, and roots were dried in an oven with air circulation at 65°C for 72 h. Plagiotropic branches are the reproductive side branches where the beans are produced. After drying, dry mass (DM) was obtained for each tissue, and the materials were finely milled in a Wiley mill.

### Phosphorus concentration and P content

The P concentration in roots, stem, branches, mature, and young leaves were determined by X-ray fluorescence spectroscopy of dispersive energy (EDXRF). Briefly, 1 g of dried plant material was packed into a polyethylene cup of 20 mm internal diameter and covered with 6-μm-thick polypropylene film (Mylar®). The samples were irradiated in triplicate for 300 s under vacuum using an energy dispersive X-ray fluorescence spectrometer (Shimadzu EDX-720, Shimadzu, Sao Paulo, SP, Brazil). The intensity of element Kα counts per second (cps/μA) was obtained from the sample X-ray spectrum deconvolution, and P concentration calculated, according methodology proposed by Tezotto et al. (2013). The P content was calculated using the P concentration and DM of each plant tissue.

### Phosphorus use efficiency

The relative efficiency of phosphorus use (REP, %) of cultivars was calculated as the ratio between the plant DM under low Pi and DM under high Pi, as described by Ozturk et al. (2005):

$$\begin{array}{l} REP=\left(\frac{DM_{low\;Pi}}{DM_{high\;Pi}}\right)x100 \end{array}$$

The agronomic P use efficiency (APE, g DM g^−1^ Pi) was obtained by expression adapted from Oliveira et al. (1987):

$$\begin{array}{l} APE=\frac{\left(DM_{high\;Pi}-DM_{low\;Pi}\right)}{\begin{array}{c} difference\;in\;the\;total\;available\;Pi\;between\\ high\;Pi\;and\;low\;Pi\;treatments \end{array}} \end{array}$$

The APE and DM at low Pi were used to separate the cultivars into different categories, as described previously (Gerloff, 1977): (i) efficient and non-responsive cultivars (ENR); (ii) efficient and responsive cultivars (ER); (iii) non-efficient and responsive cultivars (NER); and (iv) non-efficient and non-responsive cultivars (NENR).

P use efficiency was separated into P uptake efficiency (PUpE, mg P g^−1^ Pi) and P utilization efficiency (PUtE, g DM g^−1^ P). The PUpE was obtained by the ratio of P uptake in plant and the amount of Pi applied and PUtP represents the production of plant biomass per unit P accumulated in the plant:

$$\begin{array}{l} PUpE_{high\;Pi}=\frac{\left({\left[P\right]}_{high\;Pi}xDM_{high\;Pi}\right)}{Pi_{applied}}\text{or}\\ PUpE_{low\;Pi}=\;\frac{\left({\left[P\right]}_{low\;Pi}xDM_{low\;Pi}\right)}{Pi_{applied}}\\ PUtE_{high\;Pi}=\frac{DM_{high\;Pi}}{\left({\left[P\right]}_{high\;Pi}xDM_{high\;Pi}\right)}\text{or}\\ PUtE_{low\;Pi}=\;\frac{DM_{low\;Pi}}{\left({\left[P\right]}_{lowPi}xDM_{low\;Pi}\right)} \end{array}$$

The physiological P use efficiency (PPUE, (g^2^ DM g^−1^ P)) corresponds to DM produced for a given plant P concentration (Hammond et al., 2009).

$$\begin{array}{l} PPUE_{high\;Pi}=\frac{DM_{high\;Pi}}{{\left[P\right]}_{high\;Pi}}\;\text{or }PPUE_{low\;Pi}=\frac{DM_{low\;Pi}}{{\left[P\right]}_{low\;Pi}} \end{array}$$

### Statistical analyses

Data were analyzed by analysis of variance using SAS (SAS Institute INC., Cary, NC, USA) and means were compared by Tukey test at *P* < 0.05. The relationship between dry mass and relative efficiency of P were analyzed by regression analysis. Correlations between the measured data were performed using the Pearson correlation coefficient in GenStat (64-bit Release 16.1, VSN International Ltd., Hemel Hempstead, UK). Significant correlations were identified at a significance level of *P* < 0.05.

## Results

### Low Pi availability reduces coffee growth and development

**Figure 1:**
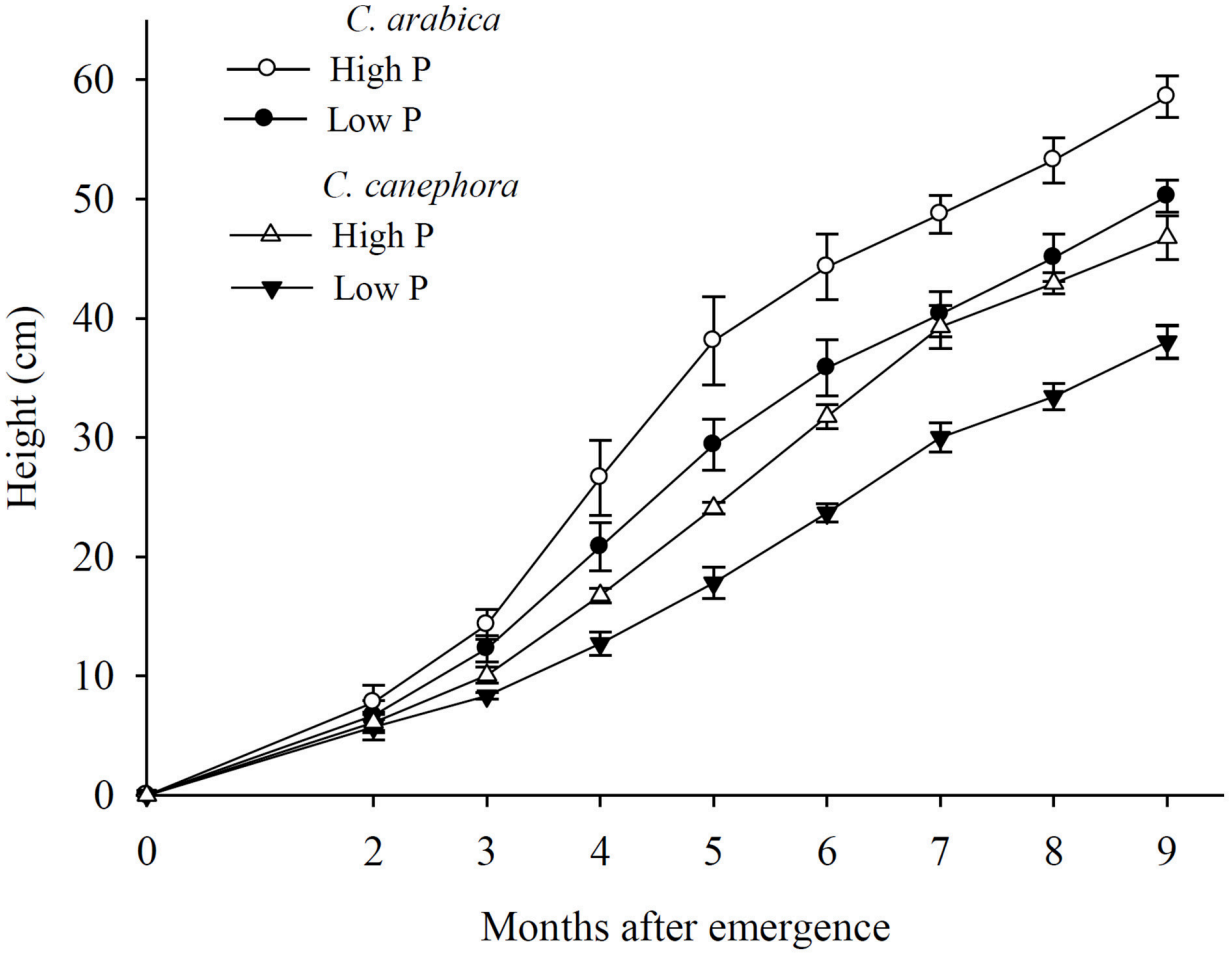
**Average height of seedlings of *Coffea arabica* (circles) and *C. canephora* (triangles) grown at low P (8 mg dm^−3^ resin P, closed symbols) and high P (120 mg dm^−3^ resin P, open symbols)**. Plants were grown under glasshouse conditions from July 2010 to August 2011 in Piracicaba, São Paulo State, Brazil. Plant height was measured from the base to the apex of the orthotropic branch.

Coffee plants grown at low Pi showed symptoms of nutrient deficiency. As the Pi deficiency progressed, mature leaves became chlorotic with necrotic lesions on the leaf apex, as observed by Ozturk et al. (2005). Total plant dry mass (DM) was reduced by approximately 50% under low Pi conditions compared to high Pi conditions and the greatest DM reduction was observed in the branches that accumulated only 43% of the DM at high Pi (Table 1). Cultivars of *C. canephora* produced more DM in the roots, regardless of the Pi treatment, compared to cultivars of *C. arabica*. Consequently, cultivars of *C. canephora* had higher root/shoot ratios (Table 1). Growth rates of plants at high Pi were higher than those at low Pi until the 5th month for *C. arabica* and the 6th month for *C. canephora* (Supplementary Table 3). After this period, growth rates were similar regardless of the Pi supply. The growth rate reduced after 6 months in both species in both Pi supplies, due to temperature reduction after April. Plant height of *C. arabica* was superior to that of *C. canephora* 9 months after emergence, at both Pi supplies (Figure 1). At the end of the experiment, the cultivars of *C. arabica* showed, on average, between 4 and 5 pairs of branches, while cultivars of *C. canephora* had two pairs of branches, attributed to the genetic characteristics of these species.

**Table 1:** **Means of dry mass of shoot and roots and ratio root/shoot of 21 cultivars of *C. arabica* and 4 cultivars of *C. canephora***.

**P Treatment**	**Species**	**Root (g)^a^**	**Shoot (g)^a^^,^^b^**	**Total (g)^a^**	**Ratio Root/Shoot^a^**
Low Pi	*C. arabica*	6.2 Bb	23.0 Ba	29.3 Bb	0.27 Ab
	*C. canephora*	7.3 Ba	18.0 Bb	25.4 Bb	0.40 Aa
High Pi	*C. arabica*	10.9 Ab	37.9 Aa	49.2 Aa	0.29 Ab
	*C. canephora*	13.4 Aa	32.8 Aa	46.3 Aa	0.43 Aa
F-test:		54.9^*^	60.4^*^	62.7^*^	22.0^*^
Species (S)		31.3^*^	15.1^*^	2.56^ns^	145.8^*^
Level of P (P)		230.0^*^	208.0^*^	222.5^*^	0.0^ns^
S x P interaction:		6.5^*^	0.7^ns^	0.2^ns^	1.0^ns^

a Values followed by the same capital letters vertically do not differ significantly (P > 0.05) between treatments (low and high Pi) and values followed by the same lowercase letters do not differ significantly (P > 0.05) between species (C. arabica and C. canephora).

b Shoot = stem + branches + mature leaves + young leaves.

* significant effect; ns, not significant.

### Coffee plant P concentration was greater in cultivars of *C. canephora*

Highest plant P concentration and content occurred in plants supplied with high Pi (Table 2). Average plant P concentrations were higher in *C. canephora* species, at both low and high Pi. Plant P content did not differ significantly between the species in low Pi, but it was significantly higher in plants of *C. canephora* at high Pi.

**Table 2:** **Plant P concentration and P content in coffee cultivars grown at low and high Pi**.

**Cultivars**	**P concentration (g P kg**^**−1**^ **DM)**		**P content (mg plant**^**−1**^**)**	
	**Low Pi**	**High Pi**	**Low Pi**	**High Pi**
***Coffea arabica***				
Typica	0.62	1.28	15.3	68.0
Bourbon Vermelho	0.65	1.07	15.9	55.9
Bourbon Amarelo	0.66	1.12	21.5	55.8
Mundo Novo	0.64	1.29	17.7	70.3
Acaiá	0.63	1.18	19.3	61.4
Caturra Vermelho	0.74	1.42	22.0	67.9
Caturra Amarelo	0.70	1.46	21.5	64.6
Catuaí Vermelho	0.69	1.35	20.1	60.6
Catuaí Amarelo	0.66	1.40	14.8	64.8
Icatu Precoce	0.63	1.20	21.5	61.7
Ouro Verde	0.82	1.39	21.6	62.1
Obatã	0.69	1.24	18.4	49.2
Tupi	0.65	1.34	19.3	65.3
E 534 Kaffa	0.66	1.18	16.9	62.7
E 208 Illubabor	0.68	1.05	19.1	50.6
E 22 Sidamo	0.61	1.03	18.1	54.8
E 16 Shoa	0.64	1.17	19.4	64.8
E 12 Harar	0.65	1.14	22.7	60.5
Jimma Tane	0.69	1.22	15.9	53.0
Geisha	0.63	1.06	25.7	51.9
Iemen	0.64	1.19	20.0	63.5
Average^a^	0.67Bb	1.23Ab	19.4Ba	60.4Ab
***Coffea canephora***				
Apoatã	0.90	1.92	23.0	86.4
Robusta	0.77	1.60	22.0	77.0
Bukobensis	0.83	1.45	22.8	63.5
Guarini	0.88	1.96	16.5	92.3
Average^a^	0.85Ba	1.74Aa	21.1Ba	79.8Aa
*F*-test: 19,89	29.58^*^		16.18^*^	
Cultivars (C):	12.32^*^		1.78^*^	
Level of P (P)	1172.40^*^		746.92^*^	
Interaction C × P	3.83^*^		1.72^*^	
Least significant difference:	0.14	0.45	10.3	40.6

a Values followed by the same capital letters do not differ significantly (P > 0.05) between treatments and values followed by the same lowercase letters do not differ significantly (P > 0.05) between species.

There was significant variation in plant P concentration and P content among the cultivars (Table 2). The P concentration ranged from 0.61 g kg^−1^ (E22 Sídamo) to 0.90 g kg^−1^ (Apoatã) in low P, and from 1.03 g kg^−1^ (E22 Sídamo) to 1.96 g kg^−1^ (Guarini) at high Pi. In turn, P content ranged from 14.8 g plant^−1^ (Catuaí Amarelo) to 25.7 g plant^−1^ (Geisha) in low Pi, and from 49.2 g plant^−1^ (Obatã) to 92.3 g plant^−−1^ (Guarini) in high P, with an increase of between 160 and 459% with Pi supply (Table 2).

Young leaves showed on average the highest P concentration, followed by mature leaves, stem+branches and roots, in both species at both Pi supplies. Phosphorus concentrations in plant tissues varied significantly among the cultivars. At low Pi, P concentration in roots ranged from 0.43 g kg^−1^ (Catuaí Amarelo) to 0.60 g kg^−1^ (Acaiá), stem+branches from 0.43 g kg^−1^ (Geisha) to 0.89 g kg^−1^ (Apoatã); in mature leaves from 0.62 g kg^−1^ (Acaiá) to 0.99 g kg^−1^ (Guarini) and in young leaves from 0.91 g kg^−1^ (E22 Sídamo, Tupi) to 1.85 g kg^−1^ (Guarini). At high Pi, P concentration in roots ranged from 0.73 g kg^−1^ (Bourbon Vermelho, Geisha, E208 Ilubabor) to 1.00 g kg^−1^ (Bukobensis), stem+branches from 0.73 g kg^−1^ (E22 Sídamo) to 2.28 g kg^−1^ (Guarini), in mature leaves from 1.17 g kg^−1^ (E22 Sídamo) to 2.14 g kg^−1^ (Guarini) and in young leaves from 1.68 g kg^−1^ (Geisha) to 2.96 g kg^−1^ (Guarini; Table 3).

**Table 3:** **P concentration in roots, stem+branches, mature leaves, and young leaves of coffee cultivars in low and high P**.

	**Roots**		**Stem+branches**		**Mature leaves**		**Young leaves**	
	**Low Pi**	**High Pi**	**Low Pi**	**High Pi**	**Low Pi**	**High Pi**	**Low Pi**	**High Pi**
	**g P kg**^**−1**^ **DM**							
***Coffea arabica***								
Typica	0.49	0.74	0.47	1.10	0.67	1.59	0.95	1.88
Bourbon V.	0.56	0.73	0.49	0.79	0.71	1.34	0.95	1.97
Bourbon A.	0.53	0.78	0.47	0.80	0.73	1.34	1.07	1.87
Mundo Novo	0.54	0.89	0.46	0.97	0.68	1.69	1.04	1.88
Acaiá	0.60	0.95	0.46	0.81	0.62	1.50	0.99	1.91
Caturra V.	0.55	0.92	0.53	1.24	0.8	1.69	1.14	1.99
Caturra A.	0.54	0.87	0.53	1.16	0.75	1.78	1.11	2.26
Catuaí V.	0.51	0.86	0.51	1.14	0.75	1.48	1.13	2.03
Catuaí A.	0.43	0.82	0.50	1.16	0.71	1.74	1.08	2.06
Icatu Precoce	0.52	0.79	0.46	0.83	0.69	1.54	0.99	2.04
Ouro Verde	0.60	0.94	0.62	1.16	0.85	1.56	1.27	2.13
Obatã	0.51	0.82	0.53	1.03	0.71	1.31	1.06	1.90
Tupi	0.55	0.86	0.57	1.37	0.63	1.40	0.91	1.91
E 534 Kaffa	0.53	0.96	0.46	0.97	0.72	1.35	1.14	1.93
E 208 Illubabor	0.53	0.73	0.48	0.80	0.78	1.25	1.12	1.81
E 22 Sidamo	0.50	0.81	0.49	0.73	0.63	1.17	0.91	1.79
E 16 Shoa	0.51	0.90	0.45	0.87	0.75	1.42	1.02	1.96
E 12 Harar	0.54	0.87	0.45	0.90	0.75	1.41	1.06	1.74
Jimma Tane	0.46	0.86	0.55	0.96	0.72	1.43	1.27	1.93
Geisha	0.56	0.73	0.43	0.85	0.74	1.24	1.07	1.68
Iemen	0.50	0.81	0.48	1.08	0.74	1.38	1.05	1.88
Average^a^	0.53	0.84	0.49	0.99	0.72	1.46	1.06	1.93
	Ba	Ab	Bb	Ab	Bb	Ab	Bb	Ab
***Coffea canephora***								
Apoatã	0.51	0.97	0.89	2.20	0.95	2.01	1.68	2.91
Robusta	0.50	0.85	0.74	1.87	0.88	1.88	1.25	2.46
Bukobensis	0.57	1.00	0.84	1.81	0.80	1.42	1.63	2.32
Guarini	0.49	0.97	0.67	2.28	0.99	2.14	1.85	2.96
Average^a^	0.52	0.95	0.79	2.04	0.91	1.86	1.60	2.66
	Ba	Aa	Ba	Aa	Ba	Aa	Ba	Aa
*F*-test:	15.98^*^		31.54^*^		17.29^*^		22.58^*^	
Cultivars (C)	252.00^*^		26.14^*^		4.92^*^		11.60^*^	
Level of P (P)	711.71^*^		802.33^*^		745.68^*^		868.11^*^	
C × P interaction:	1.66^*^		9.55^*^		1.88^*^		1.81^*^	
Least significant difference	0.17	0.27	0.19	0.53	0.18	0.72	0.39	0.74

a Values followed by the same capital letters do not differ significantly (P > 0.05) between treatments and values followed by the same lowercase letters do not differ significantly (P > 0.05) between species.

### Coffee cultivars vary in their P utilization - *C. canephora* is more efficient in P uptake and *C. arabica* is more efficient in P utilization

The relative efficiency (REP) in biomass accumulation, obtained by the ratio between DM at low Pi and DM at high Pi, was 61% on average for cultivars of *C. arabica* and 55% on average for cultivars of *C. canephora* (Table 4). The variation between genotypes for DM ranged from 18.9 g plant^−1^ for cultivar Guarani to 41 g plant^−1^ for cultivar Geisha at low Pi and 39.8 g plant^−1^ for cultivar Obatã to 55.4 g plant^−1^ for cultivar E 16 Shoa at high Pi.

**Table 4:** **Dry mass (DM), relative efficiency P (REP), and agronomic P use efficiency (APE) of 25 cultivars and their respective groups: efficient responsive (ER), efficient non-responsive (ENR), non-efficient responsive (NER) and non-efficient non-responsive (NENR) defined in Figure 2**.

**Cultivars**	**Dry mass (g plant**^**−1**^**)**		**REP (%)**	**APE**
	**Low Pi**	**High Pi**		**(g DM g^−1^ Pi)**
**ER**				
E 16 Shoa	30.25	55.39	55	24.94
E 22 Sidamo	30.12	53.45	56	23.15
Iemen	31.62	53.37	59	21.58
Acaiá	31.02	51.84	60	20.66
**ENR**				
Tupi	29.49	49.30	60	19.65
Robusta	28.90	46.64	62	17.6
Caturra Vermelho	29.62	47.33	63	17.57
E 12 Harar	35.09	52.56	67	17.33
Bourbon Amarelo	32.65	49.22	66	16.43
Icatu Precoce	34.34	50.55	68	16.08
Catuaí Vermelho	29.20	44.38	66	15.05
Caturra Amarelo	30.81	44.23	70	13.31
Geisha	40.97	49.39	83	8.36
**NER**				
Guarini	18.88	48.00	39	28.89
E 534 Kaffa	25.79	52.93	49	26.93
Bourbon Vermelho	24.58	51.62	48	26.83
Mundo Novo	27.56	54.48	51	26.71
Typica	25.22	51.73	49	26.29
Catuaí Amarelo	22.20	45.80	48	23.41
Jimma Tane	22.88	44.53	51	21.48
Apoatã	26.17	46.76	56	20.42
**NENR**				
E 208 Illubabor	28.05	48.09	58	19.88
Ouro Verde	26.42	43.97	60	17.41
Bukobensis	27.56	43.84	63	16.14
Obatã	26.53	39.81	67	13.17
Average—Reference values of **Figure 2**	28.64			19.28

REP was calculated as ([DM_Low Pi_/DM_High Pi_] × 100). APE was calculated how ([DM_High Pi_ – DM_Low Pi_]/difference in total Pi availability between high Pi and low Pi treatments). The data are average from five repetitions, for low Pi (8 mg Pi dm^-3^) and high Pi (120 mg Pi dm^-3^).

**Figure 2:**
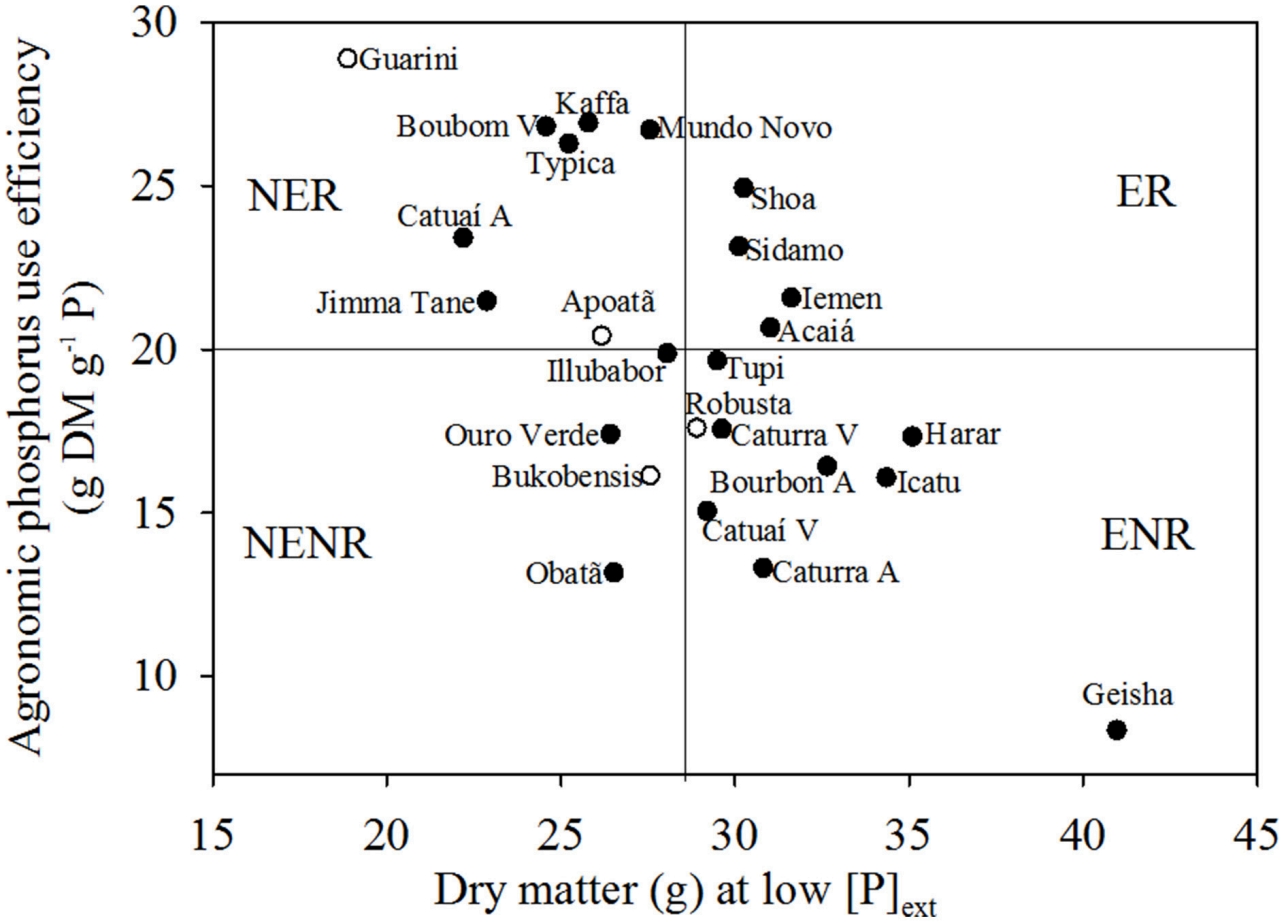
**Relationship between shoot dry matter (DM) at low P and responsiveness to P, measured as agronomic phosphorus (P) use efficiency for different coffee cultivars**. *Coffea arabica* (closed symbols) and *C. canephora* (open symbols). Solid lines represent the mean value for the axis. NER, non-efficient and responsive; ER, efficient and responsive; ENR, efficient and non-responsive; NENR, non-efficient and non-responsive.

The allocation into different groups of efficiency and response to Pi is based on the DM of the plants at low Pi (axis x) and agronomic P use efficiency (APE) index (axis y) of each cultivar (Gerloff, 1977). The average DM production of non-efficient cultivars was 25.2 g in low Pi, while efficient cultivars produced on average 31.8 g of DM in the same condition. The average APE for responsive cultivars was 24.27 g DM g^−1^ P, while the average for non-responsive cultivars was 15.99 g DM g^−1^ P. Of the 25 lines studied, only four *C. arabica* lines were classified as being efficient and responsive (Figure 2).

The P uptake and utilization efficiency varied significantly between genotypes. The PUpE, P taken up per unit of available Pi, was higher in plants grown under low Pi. Cultivars of *C. canephora* showed higher PUpE compared to *C. arabica* in both Pi treatments (Table 5). The PUpE ranged from 206 mg P g^−1^ Pi (cv. Catuaí Yellow ) to 319 mg P g^−1^ Pi (Apoatã) at low Pi and between 46 mg P g^−1^ Pi (Obatã) and 85 mg P g^−1^ Pi (Guarini) at high Pi (Table 5). The PUtE was higher in plants of *C. arabica* in both Pi treatments. At low Pi PUtE ranged from 1.12 g DM mg^−1^ P (Apoatã) to 1.66 g DM mg^−1^ P (E22 Sidamo), and at high Pi, from 0.52 g DM mg^−1^ P (Guarini) to 0.97 g DM mg^−1^ P (E22 Sidamo; Table 5).

**Table 5:** **P uptake (PUpE) (g P g^−1^ Pi) and P utilization efficiency (PUtE) (g DM g^−1^ P) and physiological P use efficiency (PPUE) (g^2^ DM mg^−1^ P) of 25 coffee cultivars grown at low and high Pi**.

**Cultivars**	**PUpE (mg P g**^**−1**^ **P)**			**PUtE (g DM mg**^**−1**^ **P)**			**PPUE (g**^**2**^ **DM mg**^**−1**^ **P)**		
	**Low Pi**		**High Pi**	**Low Pi**	**High Pi**		**Low Pi**	**High Pi**	
***Coffea arabica***									
Typica	213		63	1.64	0.79		40.6	40.5	
Bourbon Vermelho	221		52	1.56	0.95		38.0	48.3	
Bourbon Amarelo	299		52	1.53	0.90		49.7	43.9	
Mundo Novo	245		65	1.56	0.79		42.7	42.3	
Acaiá	268		57	1.60	0.86		49.5	43.9	
Caturra Vermelho	306		63	1.36	0.72		39.7	33.1	
Caturra Amarelo	298		60	1.44	0.69		44.0	30.3	
Catuaí Vermelho	280		56	1.46	0.75		42.3	32.8	
Catuaí Amarelo	206		60	1.54	0.73		33.6	32.7	
Icatu Precoce	299		57	1.61	0.84		54.7	42.0	
Ouro Verde	300		57	1.22	0.73		32.1	31.5	
Obatã	255		46	1.45	0.81		38.2	32.0	
Tupi	268		60	1.53	0.83		44.9	36.7	
E534 Kaffa	234		58	1.52	0.86		39.0	44.6	
E208 Illubabor	265		47	1.48	0.96		41.1	45.8	
E22 Sidamo	252		51	1.66	0.97		49.4	51.6	
E16 Shoa	270		60	1.56	0.87		46.9	47.4	
E12 Harar	315		56	1.54	0.88		53.8	45.9	
Jimma Tane	220		49	1.45	0.83		32.9	36.5	
Geisha	358		48	1.59	0.95		64.9	47.4	
Iemen	278		59	1.57	0.85		49.4	44.9	
Average^a^	269Ab		56Bb	1.52Aa	0.84Ba		44.2Aa	40.7Ba	
***Coffea canephora***									
Apoatã	319		80	1.12	0.53		28.9	24.3	
Robusta	306		71	1.31	0.65		37.5	29.1	
Bukobensis	317		59	1.22	0.69		33.2	30.2	
Guarini	254		85	1.15	0.52		21.3	24.4	
Average^a^	301Aa		74Ba	1.20Ab	0.60Bb		30.2Ab	27.0Bb	
Test F:	30.66^*^			45.61^*^			13.69^*^		
Cultivars (C)	2.08^*^			12.81^*^			15.10^*^		
Treatments (P)	1504.00^*^			2065.00^*^			18.46^*^		
Interaction C × P:	1.95^*^			1.40^ns^			2.52^*^		
Least significant difference	16	14		0.30		0.25	15.7		14.3

Values followed by the same capital letters do not differ significantly (P > 0.05) between treatments and values followed by the same lowercase letters do not differ significantly (P > 0.05) between species.

PPUE was higher in plants grown under Pi deficiency due to the reduced availability and uptake of Pi. Further, PPUE was higher in plants of the species *C. arabica* under both Pi treatments. PPUE varied among genotypes, ranging from 21.3 (Guarini) to 64.9 g^2^ DM mg^−1^ P (Geisha) at low Pi, and from 24.4 (Guarini) to 51.6 g^2^ DM mg^−1^ P (E22 Sidamo) under high Pi (Table 5).

Significant correlations between biomass, P uptake and measures of PUE were observed within treatments (Supplementary Table 4). At low Pi, significant positive correlations between PPUE and the P content of shoots, mature leaves and stems was observed, but no significant correlations were observed between PPUE and the P content of young leaves or roots. At low Pi, the root to shoot ratio was significantly (*P* = 0.0012) positively correlated with PUpE, but significantly (*P* = < 0.001) negatively correlated with PUtE. Root to shoot ratio was also significantly positively correlated with aboveground tissue P concentrations under both low and high Pi supplies. Root to shoot ratio was significantly (*P* = < 0.001) negatively correlated with both PUtE and PPUE under high Pi supply (Supplementary Table 4B).

## Discussion

In the current study, we assessed 21 cultivars of *C. arabica* and four cultivars of *C. canephora* and observed a wide variation of different components of PUE and growth traits in response to soil Pi availability. Interestingly, at the species level, cultivars of *C. canephora* showed higher PUpE compared to *C. arabica* in both Pi treatments and cultivars of *C. arabica* showed higher PUtE compared to *C. canephora* in both Pi treatments (Table 5). Root to shoot ratios of coffee cultivars were also positively correlated with PUpE at low soil Pi availability, but negatively correlated with PUtE (Supplementary Table 4).

The coffee varieties differed in growth and DM production in low Pi and in response to Pi supply (Table 4). The REP among the cultivars ranged from 39 (cv Guarini) to 83% (cv Geisha), i.e., the DM production in low Pi in cultivar Guarini was greatly reduced, while in Geisha, there was a slight reduction in DM between high and low Pi treatments (Table 4). Previous breeding efforts have focused on increasing grain or DM production. Since these are used in all indexes to calculate P utilization efficiencies, increased grain or DM biomass with or without changes in P concentrations in tissues can result in unintentional improvements in PUE (Hammond et al., 2009). The genotypes with greater REP indexes produced more biomass under low Pi, which indicates that DM under low Pi is a good parameter for studies on P efficiency, which was also observed by Ozturk et al. (2005) in wheat genotypes. However, DM or grain production is not sufficient for the understanding of processes that determine the PUEs of plants or of the capacity of the plant to grow productively under low Pi availability.

Using the biomass response of plants to Pi availability, plants can be grouped into “efficient” and “inefficient” based on the ability of cultivars to convert nutrients into dry matter (Vose, 1987). Furthermore, they may be grouped as “responsive” and “non-responsive” based on the plants biomass response to the addition of the nutrient (Fageria and Baligar, 1993), with plants having above average APE values grouped as “responsive” and plants having below average APE values grouped as “non-responsive” (Figure 2). Gerloff (1977) conducted the first study classifying plants into four groups in terms of efficiency and response to nutrient supply: (i) efficient and responsive - plants that produce above average biomass at lower nutrient concentrations and respond to nutrient addition; (ii) inefficient and responsive—plants that produce less than average biomass at lower nutrient concentrations but still respond to nutrient addition; (iii) efficient and non-responsive—plants that produce above average biomass at lower nutrient concentrations, but do not respond to the addition of nutrients; (iv) inefficient and non-responsive—plants that produce less than average biomass at lower nutrient concentrations, which do not respond to nutrient addition. This is the first study to use these criteria to characterize coffee cultivars for their efficient use of available Pi and response to the addition of Pi. It clearly identifies four cultivars that are efficient and responsive to Pi supply, all of which are *C. arabica.* These varieties are most likely to perform well under low Pi conditions and would provide useful genetic material in the breeding of new varieties with improved PUE.

Among the DM parameters, the root DM showed a significant difference between species. Plants of *C. canephora* have a longer root system, in both conditions of P supply (Table 1), which may explain the increased P uptake efficiency in the genotypes of this species (Table 5). This species has a more robust and vigorous root system, the reason why it is often used as a graft for plants of *C. arabica*. The evolution of *C. canephora* exposed to sunlight and in a region with an average temperature of 23°C led to greater development of the root system to provide more water for transpiration to regulate leaf temperature. Another aspect that reinforces this statement is the presence of twice as many stomata per square millimeter of leaf area in relation to cultivars of *C. arabica* (Voltan et al., 1992). Phosphate movement through the soil to the root surface is governed by diffusion, rather than mass flow. In low Pi soils, cultivars that have a larger root system are more likely to intercept and acquire P, as occurred with the cultivars of *C. canephora*. This is also reflected in the significant positive correlation between root to shoot ratio and aboveground tissue P concentrations (Supplementary Table 4).

Low soil Pi availability resulted in a significant reduction of P concentration and content of the tissues studied. The P concentration in the roots, stem+branches, mature leaves and young leaves under low Pi were 40–60% lower when compared to high Pi supply. The P content was 20–40% lower under low Pi when compared to high Pi supply.

Cultivars of *C. canephora* showed higher P concentration in tissues, in both Pi treatments (Table 2), which can be explained by the greater efficiency of Pi uptake by *C. canephora* cultivars under both high and low Pi (Table 5). Of the total P accumulated by cultivars of *C. arabica* under low Pi supply, 36% was in mature leaves, 26% in young leaves, 21% in stem+branches and 17% in the roots. The total P accumulated in *C. canephora* under low P supply was 43% in mature leaves, 20% in young leaves, 19% in stem+branches and 18% in roots. While the young and growing tissues are the main sinks for P, mature leaves still contain a significant proportion of P accumulated by coffee, with about 40% of accumulated P, even under conditions of P deficiency. Bragança et al. (2008) observed P contents of 33% in stem+ orthotropic branches, 24% in leaves, 16% in fruits, 15% in roots, and 12% in plagiotropic branches of plants of *C. canephora* in production. Correa et al. (1985) found greater P content in leaves (33%), followed by fruits (27%), branches (18%), stem (13%), and roots (9%). These results are consistent with those observed here. At high Pi, cultivars of *C. canephora* accumulated more P, compared to those of *C. arabica*. This higher P content in cultivars of *C. canephora* might be associated with the greater root biomass observed in the species.

The PPUE represents the DM production per P unit present in the same mass portion. In this experiment, the PPUE showed positive correlations with the biometric parameters such as height, diameter, number of leaves, branches, and total DM in plants, all of which are related to plant DM, a significant component in the calculation of PPUE. In turn, the P concentration correlated negatively with PPUE, i.e., plants that concentrated more P in tissues showed reduced PPUE. The cultivars with higher PPUE feature lower P content in mature leaves and stem+branches compared to cultivars with lower PPUE (Supplementary Table 4). This lower P concentration in mature leaves and stem+branches could be attributed to P remobilization from mature tissues and storage organs to growing tissues, such as young leaves and roots or a consequence of initial distribution during uptake. This requires further investigation and may provide an interesting trait for breeding new cultivars with improved PPUE.

At low Pi supply, the cultivars Geisha, Icatu Precoce, and E12 Harar had the highest PPUE and Guarani, Apoatã, Bukobensis, and Ouro Verde had the lowest PPUE. This classification is consistent with the classification of Figure 2, which separates all the cultivars by their efficient use of P and responsiveness to available Pi. In this case, the cultivars with higher PPUE were also classified as efficient and had lower P concentrations in stems + branches and mature leaves compared to cultivars with lower PPUE. The cultivars Obatã, Ouro Verde, and Bukobensis, classified as inefficient and unresponsive, had lower PUtE and PPUE. Cultivar Obatã still showed low PUpE in high Pi supply, which explains the low responsiveness to added Pi.

In summary, significant variation in PUE and its component traits was observed across a wide genepool of coffee cultivars. Significantly, at the species level, cultivars of *C. canephora* showed higher PUpE compared to *C. arabica* and cultivars of *C. arabica* showed higher PUtE compared to *C. canephora*. Positive correlations between PUpE and root to shoot ratio were also observed across all cultivars at low Pi supply, suggesting this or root related traits are valuable targets for improving the PUpE of coffee in Pi limited soils. These data provide information about individual cultivars and their suitability for growing under low Pi availabilities and identifies cultivars with contrasting PUE that may be suitable for use in breeding programs to improve these traits in new cultivars. The correlation between roots and PUpE suggests an important role for roots in the acquisition of Pi by coffee and requires further research to identify specific traits controlling this.

## Author contributions

AN, JF and TT conceived and designed the experiments. AN and TT conducted experiments. AN, JH, TT, and JF contributed to the analysis and interpretation of the data. HC contributed to the statistical analysis of the data. AN, JF, JH, and TT wrote the manuscript; all authors contributed to the discussion and approved the final manuscript.

## Funding

We acknowledge funding from São Paulo Research Foundation (FAPESP) Resource for Project Execution Grant number 2010/11744-2 and Scholarship Grant number 2010/11745-9.

### Conflict of interest statement

The authors declare that the research was conducted in the absence of any commercial or financial relationships that could be construed as a potential conflict of interest. The reviewer BD and handling Editor RB declared their shared affiliation, and the handling Editor states that the process nevertheless met the standards of a fair and objective review.
