# Supplementary material for: Analysis of Phosphorus Use Efficiency Traits in Coffea Genotypes Reveals Coffea arabica and Coffea canephora Have Contrasting Phosphorus Uptake and Utilization Efficiencies
Source: Front Plant Sci. 2016 Mar 31;7:408. doi: 10.3389/fpls.2016.00408 (PMC4814561; doi:10.3389/fpls.2016.00408)
Supplement: Supplementary file 1 [file Table1.DOCX]

| Supplementary Table 1. Identity and origin of genetic material used in the experiment. | | | | | |
| --- | --- | --- | --- | --- | --- |
|  | **Species** | **Identification** | **IAC Registration** | **Cultivar type** | **Country** |
| 1 | *C. arabica* | Typica |  | No selection | Brazil |
| 2 | *C. arabica* | Bourbon Vermelho | IAC 662 | Improved/Cultivar | Brazil |
| 3 | *C. arabica* | Bourbon Amarelo | IAC J19 | Improved/Cultivar | Brazil |
| 4 | *C. arabica* | Mundo Novo | IAC 376-4 | Improved/Cultivar | Brazil |
| 5 | *C. arabica* | Acaiá | IAC 474-19 | Improved/Cultivar | Brazil |
| 6 | *C. arabica* | Caturra Vermelho | IAC 477 | Improved/Cultivar | Brazil |
| 7 | *C. arabica* | Caturra Amarelo | IAC 476 | Improved/Cultivar | Brazil |
| 8 | *C. arabica* | Catuaí Vermelho | IAC 144 | Improved/Cultivar | Brazil |
| 9 | *C. arabica* | Catuaí Amarelo | IAC 62 | Improved/Cultivar | Brazil |
| 10 | *C. arabica* | Icatu Precoce | IAC 3282 | Improved/Cultivar | Brazil |
| 11 | *C. arabica* | Ouro Verde | IAC H5010-5 | Improved/Cultivar | Brazil |
| 12 | *C. arabica* | Obatã | IAC 1669-20 | Improved/Cultivar | Brazil |
| 13 | *C. arabica* | Tupi | IAC 1669-33 | Improved/Cultivar | Brazil |
| 14 | *C. arabica* | E 534 - Kaffa | IAC 2197 | No selection | Ethiopia |
| 15 | *C. arabica* | E 208 - Illubabor | IAC 2092 | No selection | Ethiopia |
| 16 | *C. arabica* | E 22 Sidamo | IAC 2032 | No selection | Ethiopia |
| 17 | *C. arabica* | E 16 - Shoa | IAC 2027 | No selection | Ethiopia |
| 18 | *C. arabica* | E 12 - Harar | IAC 2026 | No selection | Ethiopia |
| 19 | *C. arabica* | Jimma Tane | IAC 1124-2 | Improved/exotic cultivar | Ethiopia |
| 20 | *C. arabica* | Geisha | IAC 2210 | Improved/exotic cultivar | Ethiopia |
| 21 | *C. arabica* |  |  | Improved/exotic cultivar | Yemen |
| 22 | *C. canephora* | Apoatã | IAC 2258 | Improved/Cultivar | Brazil |
| 23 | *C. canephora* | Robusta | IAC 2292 | No selection | Brazil |
| 24 | *C. canephora* | Bukobensis | IAC 451 Col 2 | No selection | Brazil |
| 25 | *C. canephora* | Guarini |  | No selection | Brazil |
