# Supplementary material for: Analysis of Phosphorus Use Efficiency Traits in Coffea Genotypes Reveals Coffea arabica and Coffea canephora Have Contrasting Phosphorus Uptake and Utilization Efficiencies
Source: Front Plant Sci. 2016 Mar 31;7:408. doi: 10.3389/fpls.2016.00408 (PMC4814561; doi:10.3389/fpls.2016.00408)
Supplement: Supplementary file 2 [file Table2.DOCX]

| Supplementary Table 2. Soil chemical and physical analysis after addition of nutrients. | | | | |
| --- | --- | --- | --- | --- |
| Soil Property | Extractant | Unit | Treatment | |
|  |  |  | Low Pi | High Pi |
| pH^1^ | CaCl_2_ |  | 5 | 4.8 |
| OM^2^ |  | g dm^-3^ | 11 | 10 |
| P-resin | cation exchange resin | mg dm^-3^ | 8 | 120 |
| K | cation exchange resin | mmol_c_ dm^-3^ | 4 | 4 |
| Ca | cation exchange resin | mmol_c_ dm^-3^ | 24 | 22 |
| Mg | cation exchange resin | mmol_c_ dm^-3^ | 11 | 10 |
| Al |  | mmol_c_ dm^-3^ | 0 | 1 |
| CTC^3^ |  | mmol_c_ dm^-3^ | 59 | 61 |
| Cu | DTPA^4^ | mg dm^-3^ | 1 | 1 |
| Fe | DTPA^4^ | mg dm^-3^ | 42 | 56 |
| Zn | DTPA^4^ | mg dm^-3^ | 3 | 3 |
| Mn | DTPA^4^ | mg dm^-3^ | 13 | 16 |
| B | hot water | mg dm^-3^ | 0.23 | 0.29 |
| Soil texture |  |  | Mean | |
| Clay |  | g kg^-1^ | 189 | |
| Silt |  | g kg^-1^ | 41 | |
| Total Sand |  | g kg^-1^ | 770 | |
| Thick Sand |  | g kg^-1^ | 320 | |
| Fine Sand |  | g kg^-1^ | 450 | |
| ^1^pH - hydrogen potential; ^2^OM – organic matter; ^3^CTC - cation exchange capacity; ^4^DTPA - diethylene triamine pentaacetic acid | | | | |
