# Supplementary material for: Analysis of Phosphorus Use Efficiency Traits in Coffea Genotypes Reveals Coffea arabica and Coffea canephora Have Contrasting Phosphorus Uptake and Utilization Efficiencies
Source: Front Plant Sci. 2016 Mar 31;7:408. doi: 10.3389/fpls.2016.00408 (PMC4814561; doi:10.3389/fpls.2016.00408)
Supplement: Supplementary file 3 [file Table3.DOCX]

| Supplementary Table 3. Average growth rates and emergence rate of branches from of seedlings of *C. arabica* and *C. canephora* at low and high Pi. | | | | | | | | |
| --- | --- | --- | --- | --- | --- | --- | --- | --- |
|  | Average growth rate (cm month^-1^) | | | | Branch emergence (pairs month^-1^) | | | |
| Age (month) | *C. arabica* | | *C. canephora* | | *C. arabica* | | *C. canephora* | |
|  | Low Pi | High Pi | Low Pi | High Pi | Low Pi | High Pi | Low Pi | High Pi |
| 2 | 3.34b | 3.89a | 2.86a | 3.05a | - | - | - | - |
| 3 | 5.61b | 6.52a | 2.62b | 3.97a | - | - | - | - |
| 4 | 8.55b | 12.33a | 4.38b | 6.69a | 0.04b | 0.13a | 0.00a | 0.00a |
| 5 | 8.54b | 11.48a | 5.09b | 7.34a | 0.68b | 1.31a | 0.00a | 0.05a |
| 6 | 6.46a | 6.20a | 5.87b | 7.67a | 0.84a | 0.88a | 0.05a | 0.35a |
| 7 | 4.50a | 4.42a | 6.34a | 7.53a | 0.57a | 0.64a | 0.10b | 0.60a |
| 8 | 4.75a | 4.52a | 3.43a | 3.65a | 0.85b | 1.09a | 0.15a | 0.55a |
| 9 | 5.15a | 5.34a | 4.59a | 3.82a | 0.87a | 0.83a | 0.40a | 0.65a |
| **Average per month** | **5.86** | **6.84** | **4.40** | **5.46** | **0.64** | **0.81** | **0.12** | **0.37** |
| ^a^Values followed by the same capital letters horizontally do not differ significantly (P>0.05) between low and high Pi treatments within each species. | | | | | | | | |
